# Glycopyrronium 320 μg/mL in children and adolescents with severe sialorrhoea and neurodisabilities: An open‐label study extension of the SALIVA trial

**DOI:** 10.1111/dmcn.16251

**Published:** 2025-01-31

**Authors:** Pierre Fayoux, Mickael Dinomais, Helen Shaw, Frédéric Villain, Déborah Schwartz, Vincent Gautheron, Guy Letellier, Stéphane Auvin

**Affiliations:** ^1^ Department of Paediatric Otolaryngology Head Neck Surgery Jeanne de Flandre Hospital Lille France; ^2^ Université de Lille, ULR 2694–METRICS: Évaluation des Technologies de Santé et des Pratiques Médicales Lille France; ^3^ Department of Physical Medicine and Rehabilitation CHU Angers‐Les Capucins Angers France; ^4^ Proveca Ltd Manchester UK; ^5^ Kappa Santé Paris France; ^6^ Department of Pediatric Physical Medicine and Rehabilitation, CHU Saint‐Etienne and UJM Saint‐Etienne, Interuniversity Laboratory of Motricity Biology University of Lyon/Saint‐Etienne Saint‐Etienne France; ^7^ Department of Physical Medicine and Rehabilitation ESEAN‐APF Nantes France; ^8^ APHP, Service de Neurologie Pédiatrique EpiCARE ERN Membre, Hôpital Robert Debré Paris France; ^9^ Université Paris‐Cité, INSERM NeuroDiderot Paris France; ^10^ Institut Universitaire de France Paris France

## Abstract

**Aims:**

To test the long‐term efficacy, safety, and impact on quality of life (QoL) of an oral paediatric formulation of 320 μg/mL glycopyrronium in the 36‐week SALIVA (Sialanar plus orAl rehabiLitation against placebo plus oral rehabilitation for chIldren and adolescents with seVere sialorrhoeA and neurodisabilities) trial.

**Method:**

In the initial 12‐week blinded period, 87 children with neurodisabilities and severe sialorrhoea were randomized to 320 μg/mL glycopyrronium versus placebo. In the subsequent 24‐week open‐label study extension, 74 children received 320 μg/mL glycopyrronium (37 continued glycopyrronium, 37 switched from placebo).

**Results:**

The open‐label study extension population included 39 males and 35 females. The median age was 10 years 2 months (quartile 1, quartile 3: 7 years 5 months, 14 years 7 months; range: 3 years 5 months–17 years 8 months). Over 36 weeks, continued 320 μg/mL glycopyrronium resulted in a median 39‐point reduction in Drooling Impact Scale (DIS) score from baseline (quartile 1, quartile 3: −51, −21; *p* < 0.001), with an 81.1% response rate (DIS improvement ≥ 13.6 points) and a 70.3% good response rate (≥ 28 points). Improvements in the impact of drooling on QoL seen in the blinded period were sustained with continued glycopyrronium. Treatment‐related adverse events occurred most frequently during titration (0–4 weeks: 40.9%; 5–20 weeks: 32.4% in those who switched). Constipation was the most common adverse event.

**Interpretation:**

Long‐term treatment with 320 μg/mL glycopyrronium resulted in significant sustained improvements in drooling and QoL, with fewer adverse events after initial titration and overall good tolerability.

## Graphical abstract

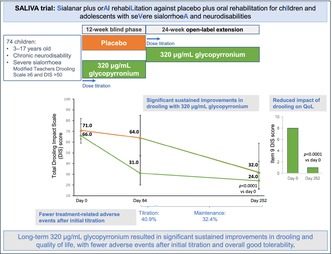

Plain language summary: https://onlinelibrary.wiley.com/doi/10.1111/dmcn.16268

**Abbreviations**
DIS: Drooling Impact Scale
DIS‐F: French version of the DIS
OLSE: open‐label study extension
QoL: quality of life
SALIVA: Sialanar plus orAl rehabiLitation against placebo plus oral rehabilitation for chIldren and adolescents with seVere sialorrhoeA and neurodisabilities

**What this paper adds**

- More than 80% of children responded to 320 μg/mL glycopyrronium over 36 weeks.
- The significant effects on quality of life seen at 12 weeks were sustained in the long term.
- Adverse events were more common during dose titration than with continued treatment.

Excessive drooling is common in children with neurodisabilities, occurring in 40% to 60% of children with cerebral palsy (CP), and is severe in 15% of cases.^1^, ^2^, ^3^ In addition to skin maceration, dehydration, aspiration, and recurrent respiratory infections, severe drooling has a substantial impact on the quality of life (QoL) of children and their families.^3^, ^4^, ^5^, ^6^, ^7^

Non‐pharmacological rehabilitations, including intraoral stimulation and oral facial exercises, are typically used initially but many children require medication to improve severe drooling. Anticholinergics, such as scopolamine patches and atropine, are often given off‐label to children with neurodisabilities in clinical practice. Botulinum neurotoxin A was licensed in Europe in 2022, with effects lasting about 16 weeks.^8^ Licensed formulations of glycopyrronium are now available: a 160 μg/mL glycopyrronium solution (equivalent to 1 mg/5 mL glycopyrronium bromide) was approved in the USA^9^ in 2010 and a formulation of 320 μg/mL glycopyrronium (equivalent to 400 μg/mL [2 mg/5 mL] glycopyrronium bromide) was approved in Europe in 2017.^10^ Paediatric formulations should be palatable, accurately titrated, and have minimal excipients;^11^ the 320 μg/mL glycopyrronium formulation was developed with this in mind. The improved bioavailability of 320 μg/mL glycopyrronium versus 160 μg/mL glycopyrronium results in a concentrated solution with 60% less volume per mg dose.^10^

The 320 μg/mL glycopyrronium formulation was recently evaluated in the initial double‐blind period of the randomized SALIVA (Sialanar plus orAl rehabiLitation against placebo plus oral rehabilitation for chIldren and adolescents with seVere sialorrhoeA and neurodisabilities) trial.^12^, ^13^ Over 12 weeks, 320 μg/mL glycopyrronium significantly improved the primary endpoint of change in Drooling Impact Scale (DIS) from baseline compared with placebo.^13^ Children and their families also reported statistically significant improvements in the extent that drooling affected their lives as well as practical benefits, for example, reduction in the number of daily bib and clothing changes. The most common adverse events with 320 μg/mL glycopyrronium were constipation (20.5%), followed by vomiting and dry mouth (both 6.8%), which occurred most frequently in the initial titration period and are consistent with the known safety profile of anticholinergics.^7^

Few trials have been conducted with glycopyrronium formulations and their duration has been short (8 weeks,^14^ 12 weeks,^15^ and 24 weeks^16^). We report the results of the 24‐week open‐label study extension (OLSE) of the SALIVA trial, which was designed to study the continued effects of longer‐term treatment with 320 μg/mL glycopyrronium on drooling, QoL, and tolerability beyond the initial 12‐week period.

## METHOD

### Trial design

As described previously,^12^, ^13^ the initial blinded period of the SALIVA trial compared the oral formulation of 320 μg/mL glycopyrronium with placebo from day 0 to day 84 (± 5 days) (EudraCT no. 2020–005534‐15) (Figure S1). All children who completed the blinded period were invited to enrol in a 24‐week OLSE. Children initially randomized to receive 320 μg/mL glycopyrronium continued on their current dose, while children randomized to receive placebo initiated 320 μg/mL glycopyrronium, with dose titration over a period of up to 4 weeks (day 84 to day 112 ± 1 day), which is consistent with the Summary of Product Characteristics of the licensed drug^10^ (Table S1). Telephone interviews were conducted every week during titration and on day 140 (± 2 days). Outpatient visits took place on day 168 (± 4 days) and at, or around, day 252 (± 7 days; study end). The protocol was approved by an independent ethics committee and the French Agence Nationale de Sécurité du Médicament.

### Trial population

Children with parental informed written consent were enrolled into the blinded period by investigators from 13 centres in France based on criteria including chronic neurodisabilities (such as CP, Angelman syndrome, Rett syndrome, epilepsy, and intellectual disability), severe sialorrhoea (defined as ≥6 on the modified Teachers Drooling Scale) and a DIS score of 50 or greater. Children were not eligible to enrol in the blinded period if they received other recent treatment for drooling, namely any anticholinergic therapy in the previous 4 weeks, botulinum neurotoxin A injection within 6 months, or surgery in the previous 12 months. Eligible children had previously received 3 months or more of non‐pharmacological rehabilitation, based on French regional recommendations,^17^ and continued to receive the same non‐pharmacological rehabilitation regimen during the blinded period and OLSE.

### Endpoints

Efficacy was assessed in the OLSE, as in the blinded period, using the validated French version of the DIS (DIS‐F).^18^ Change in total DIS score was analysed between day 0 (randomization) and day 252 (± 7 days) and between day 84 (start of the OLSE) and day 252 for children who continued to receive 320 μg/mL glycopyrronium, and for children who switched from placebo to 320 μg/mL glycopyrronium. Based on Reid et al.,^19^ the proportion of responders (DIS improvement ≥ 13.6 points) and good responders (DIS improvement ≥ 28 points) was assessed on day 252.

As in the blinded period, the QoL endpoints in the OLSE included change from day 0 to day 252 and change from day 84 to day 252 in DIS item 9 (To what extent did your child's drooling affect his or her life?) and in DIS item 10 (To what extent did your child's dribbling affect you and your family's life?). An additional QoL endpoint was changes from day 0 to day 252 in the 37‐item adult DISABKIDS questionnaire and the 12‐item DISABKIDS child version (aged ≥8 years), which is a generic QoL tool for children and adolescents with chronic diseases, regardless of pathology.^20^ DIS‐F and DISABKIDS questionnaires were completed by the investigator in an interview fashion with the same parent or carer (where possible).

As in the blinded period, adverse events were collected at every visit, and outside of visits as required, up to day 252. Data are presented for the entire 36‐week period for the continued 320 μg/mL glycopyrronium group and for day 84 to day 252 for both OLSE groups.

### Statistical methods

Due to a non‐normal distribution, even with attempted transformation, a non‐parametric Wilcoxon signed‐rank test was used to assess significant within‐group changes over time.

## RESULTS

Of the 87 children randomized, 37 in the 320 μg/mL glycopyrronium group and 39 in the placebo group completed the blinded period (Figure S2). All but two children entered the OLSE. Seventy‐four children entered the OLSE: 37 children who had been randomized to receive 320 μg/mL glycopyrronium continued on active treatment and 37 children who had previously received placebo were switched to 320 μg/mL glycopyrronium (Figure S2). Around half of the OLSE population were females (35; 47.3%) and the median age was 10 years 2 months (quartile 1 [Q1], quartile 3 [Q3]: 7 years 5 months, 14 years 7 months; range: 3 years 5 months–17 years 8 months) (Table 1). Children commonly had CP (47.3%), intellectual disability (31.1%), epilepsy (29.7%), or other neurological disorders (37.8%), with many having dual diagnoses.

**TABLE 1:** Baseline (day 0) characteristics of children included in the open‐label study extension.

	Continued 320 μg/mL glycopyrronium (*n =* 37)	Switched to 320 μg/mL glycopyrronium (*n =* 37)
Female sex, *n* (%)	14 (37.8)	21 (56.8)
Age (years:months), median (quartile 1, quartile 3)	10:4 (7:7, 14:8)	9:8 (7:4, 13:0)
Neurodisability, *n* (%)		
Cerebral palsy	15 (40.5)	20 (54.1)
Intellectual disability	11 (29.7)	12 (32.4)
Epilepsy	10 (27.0)	12 (32.4)
Other neurological disorder	19 (51.4)	9 (24.3)
Rett syndrome	1 (2.7)	0 (0)
Angelman syndrome	0 (0)	1 (2.7)
Modified Teacher's Drooling Scale score, *n* (%)		
6 = severe: drools to the extent that clothing becomes damp; occasionally	1 (2.7)	1 (2.7)
7 = severe: drools to the extent that clothing becomes damp; frequently	4 (10.8)	2 (5.4)
8 = profuse: clothing, hands, tray, and objects become wet; occasionally	6 (16.2)	2 (5.4)
9 = profuse: clothing, hands, tray, and objects become wet; frequently	26 (70.3)	32 (86.5)
Prior medical treatment for sialorrhoea,^a^ *n* (%)		
No prior medical treatment	13 (35.1)	16 (43.2)
Scopolamine only	12 (32.4)	7 (18.9)
Botulinum neurotoxin A injection only	2 (5.4)	3 (8.1)
Scopolamine patch and botulinum neurotoxin A injection	7 (18.9)	9 (24.3)
Scopolamine patch, botulinum neurotoxin A injection, and surgery	1 (2.7)	2 (5.4)
Scopolamine injection given orally	1 (2.7)	0
Scopolamine patch plus benzhexol (trihexyphenidyl)	1 (2.7)	0

^a^ Children were only eligible if they had not received any anticholinergic therapy in the previous 4 weeks, botulinum neurotoxin A within 6 months, or surgery for drooling in the previous 12 months.

On day 0, 89.2% of children had a modified Teachers Drooling Scale score of 8 or 9 indicating profuse drooling. In total, 78.4% of children were still receiving oromotor rehabilitation, with difficulties after rehabilitation (37.5%) and lack of efficacy (31.3%) cited as the most common reasons for those who discontinued. The population included both medication‐naive children (39.2%) and those who had previously received pharmacological therapy (60.8%). In total, 25.7% had received scopolamine patches only, 6.8% had received botulinum neurotoxin A injections only, and 21.6% had received a combination of scopolamine and botulinum neurotoxin A (Table 1). The mean time from the end of the other medical treatment to study treatment was 3 years 8 months (SD = 2 years 6 months) for the 39 children who received scopolamine and 2 years 1 month (SD = 1 year 8 months) for the 24 children who received botulinum neurotoxin A.

Most children completed the OLSE: four children who continued on 320 μg/mL glycopyrronium withdrew (two because of adverse events, one because of ineffective treatment, and one withdrew consent) while five children who switched to 320 μg/mL glycopyrronium withdrew (because of adverse events). At the end of the blinded phase, the median (Q1, Q3) volume received was 4.0 mL (3.2, 6.0) in the 320 μg/mL glycopyrronium group (Table 2). Doses were similar on day 168 and on day 252 in those who continued on 320 μg/mL glycopyrronium (mean [SD] = 4.7 mL [1.3] and 4.7 mL [1.2] respectively; median [Q1, Q3]: 4.5 mL (3.6, 6.0) and 5.0 mL (3.6, 6.0) respectively). There was a dose increase between day 84 and day 252 in six children who continued on 320 μg/mL glycopyrronium: to improve efficacy in four children; because of the parents' decision in one child; and accidentally in another child. There was a dose decrease between day 84 and day 252 in three children who continued on 320 μg/mL glycopyrronium: in one child because of good efficacy and because of the parents' decision in two children.

**TABLE 2:** Study treatment dosing.

	320 μg/mL glycopyrronium (*n =* 44 randomized, *n =* 44 completed), median (Q1, Q3)	Placebo (*n =* 43 randomized, *n =* 44 completed), median (Q1, Q3)
Day 0	1.1 (0.8, 1.4)	1.0 (0.8, 1.6)
Day 28	3.8 (3.0, 5.8)	4.0 (3.2, 6.0)
Day 84	4.0 (3.2, 6.0)	3.0 (1.2, 5.0)
	Continued 320 μg/mL glycopyrronium (*n =* 37 enrolled, *n =* 33 completed)	Switched to 320 μg/mL glycopyrronium (*n =* 37 enrolled, *n =* 32 completed)
Day 168	4.5 (3.6, 6.0)	4.0 (3.0, 6.0)
Day 252	5.0 (3.6, 6.0)	4.0 (3.0, 6.0)

Abbreviation: Q, quartile.

**FIGURE 1:**
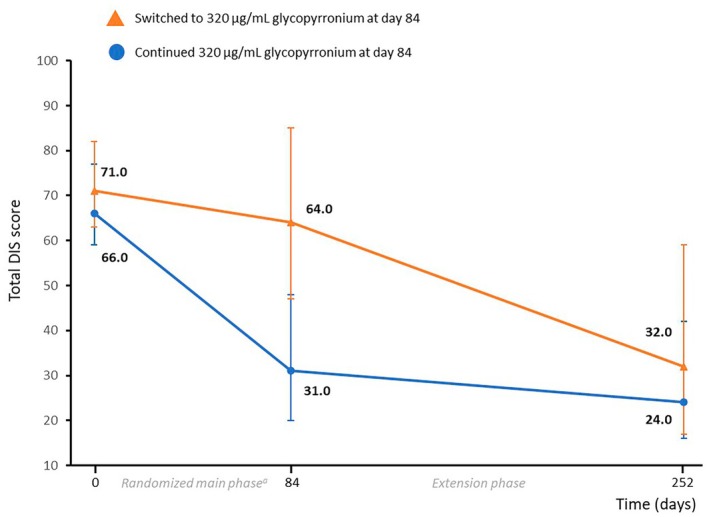
Total Drooling Impact Scale (DIS) score over time. The values represent the median (Q1, Q3). Total DIS scores range from 10 to 100, with higher scores indicating greater severity and impact. ^a^In the randomized main phase population, the median (Q1, Q3) change in total DIS score from baseline to day 84 was significantly greater with 320 μg/mL glycopyrronium vs. placebo (−29.5 [−44.5, 0] vs. −1 [−16, 5]; *p* < 0.001).^13^ Abbreviation: Q, quartile.

**FIGURE 2:**
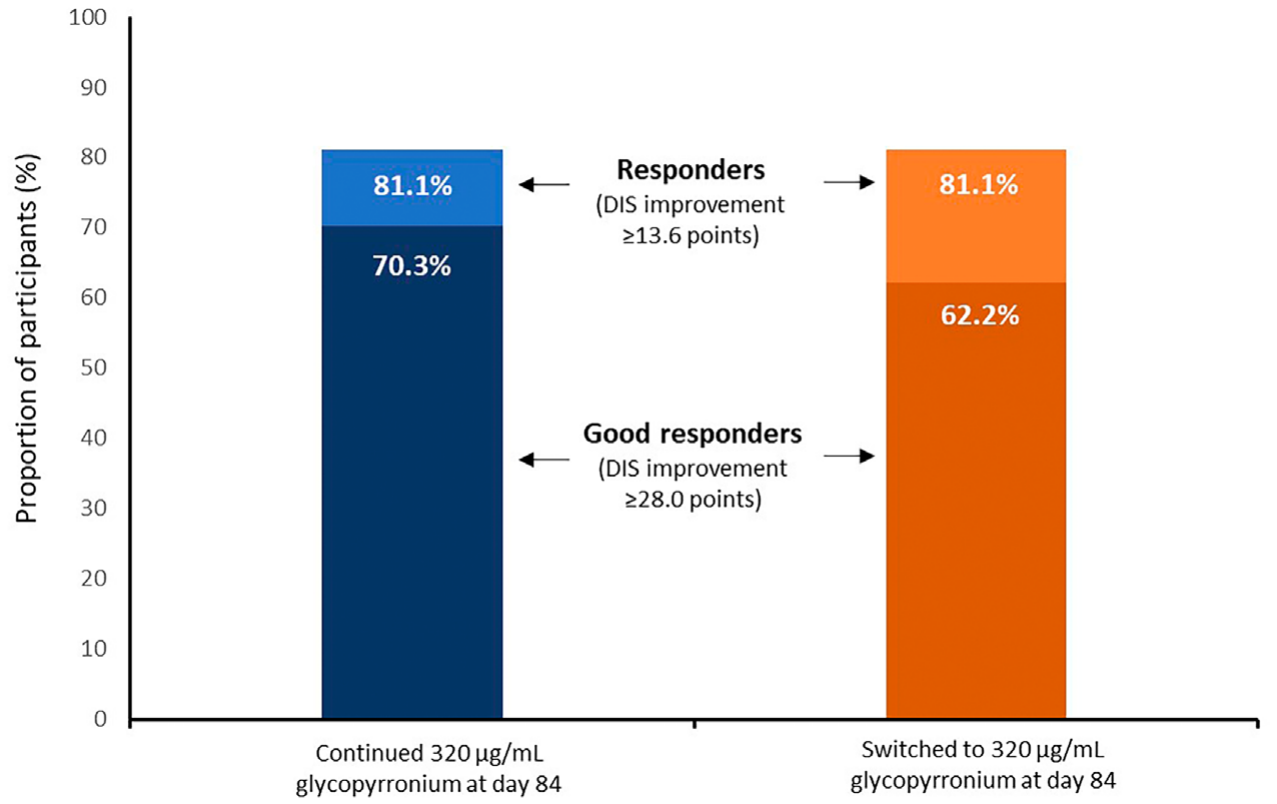
Proportion of Drooling Impact Scale (DIS) responders and good responders for the entire 36‐week study period. In the randomized main phase population, the proportions of responders and good responders on day 84 were significantly greater with 320 μg/mL glycopyrronium vs. placebo (63.6% vs. 34.9%, *p* < 0.01, and 52.3% vs. 16.3%, *p* < 0.01 respectively).^13^

Across the entire 36‐week period, the median (Q1, Q3) change in total DIS score with continued 320 μg/mL glycopyrronium was highly significant at −39 points (−51, −21; *p* < 0.001), with 30 responders (81.1%) and 26 good responders (70.3%) assessed on day 252 (Table 3, and Figures 1 and 2). Continuing treatment from day 84 to day 252 enabled a significant clinical improvement in DIS change of at least 13.6 points (response) for eight children (21.6%) and of at least 28 points (good response) for one child (2.7%).

**TABLE 3:** Total DIS score^a^.

	Continued 320 μg/mL glycopyrronium (*n =* 37 enrolled, *n =* 33 completed)	Switched to 320 μg/mL glycopyrronium (*n =* 37 enrolled, *n =* 32 completed)
Entire 36‐week period		
Day 0, median (Q1, Q3)	66.0 (59.0, 77.0)	71.0 (63.0, 82.0)
Day 252, median (Q1, Q3)	24.0 (16.0, 42.0)	32.0 (17.0, 59.0)
Median change at day 252 vs. day 0 (Q1, Q3)	−39.0 (−51.0, −21.0)	−34.0 (−47.0, −16.0)
*p*	< 0.001	< 0.001
Responders^b^ on day 252 vs. day 0, *n* (%)	30 (81.1)	30 (81.1)
Good responders^c^ on day 252 vs. day 0, *n* (%)	26 (70.3)	23 (62.2)
Open‐label study extension only		
Day 84, median (Q1, Q3)	31.0 (20.0, 48.0)	64.0 (47.0, 85.0)
Day 252, median (Q1, Q3)	24.0 (16.0, 42.0)	32.0 (17.0, 49.0)
Median change on day 252 vs. day 84 (Q1, Q3)	−2.0 (−12.0, 0)	−18.0 (−44.0, −5.0)
*p*	0.005	< 0.001
Responders^b^ on day 252 vs. day 84, *n* (%)	8 (21.6)	22 (59.5)
Good responders^c^ on day 252 vs. day 84, *n* (%)	1 (2.7)	14 (37.8)

Abbreviations: DIS, Drooling Impact Scale; Q, quartile.

^a^ Higher scores indicate greater severity and impact. ^b^DIS improvement ≥ 13.6 points. ^c^DIS improvement ≥ 28.0 points.

In participants initially on placebo who switched to 320 μg/mL glycopyrronium for the OLSE, the median change in total DIS score between day 84 and day 252 of −18 points was highly significant (−44, −5; *p <* 0.001) (Table 3). The switch to 320 μg/mL glycopyrronium for the OLSE enabled 22 children to be responders (59.5%) and 14 children to be good responders (37.8%) compared with day 84.

**FIGURE 3:**
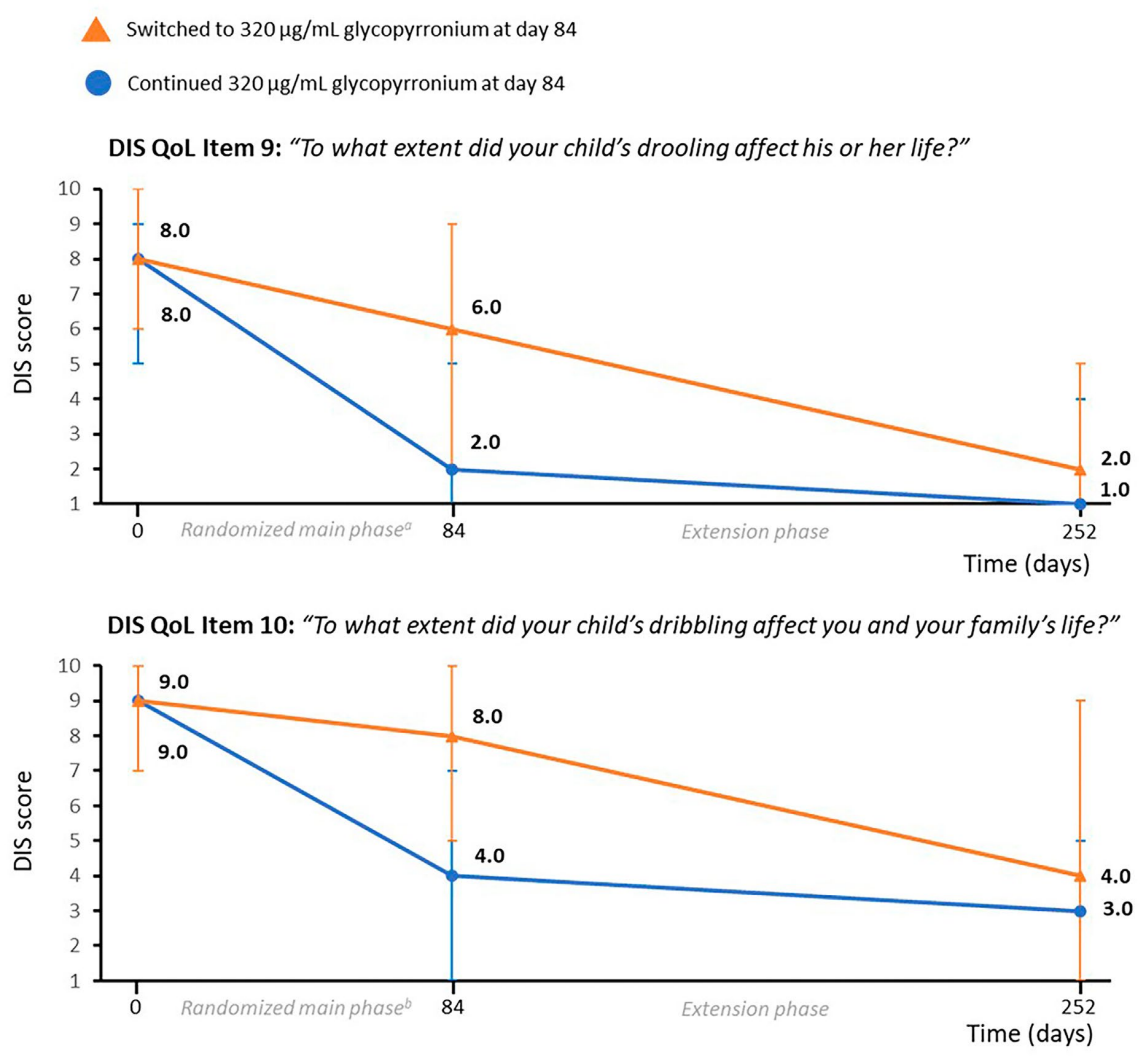
Quality of life (QoL)‐related Drooling Impact Scale (DIS) scores over time. Values represent the median (Q1, Q3). Higher DIS scores indicate greater severity and impact on QoL. Children with available measures at each time point were analysed. ^a,b^In the randomized main phase population, the median (Q1, Q3) changes in DIS scores from baseline to day 84 for items 9 and 10 were significantly greater with 320 μg/mL glycopyrronium vs. placebo (item 9: −3 [−5, 0] vs. 0 [−3, 0], *p* = 0.01; item 10: −2.5 [−7, 0] vs. 0 [−2, 0], *p* < 0.01).^13^ Abbreviation: Q, quartile.

For the DIS item 9 about the impact of drooling on a child's life, continued 320 μg/mL glycopyrronium resulted in a highly significant reduction between day 0 and day 252 (−4; −7, −1; *p* < 0.001) (Table 4 and Figure 3). In children who switched to 320 μg/mL glycopyrronium, the median DIS item 9 score improved significantly from 6 on day 84 to 2 on day 252 (median change −1; −5, 0; *p* < 0.001). Similarly, for DIS item 10 about the impact of dribbling on the child's and family's life, continued 320 μg/mL glycopyrronium resulted in a highly significant reduction between day 0 and day 252 (−5; −7, −2; *p* < 0.001) (Table 4 and Figure 3). In children who switched to 320 μg/mL glycopyrronium, there was a highly significant improvement from 8 on day 84 to 4 on day 252 (median change −2; −5, 0; *p* < 0.001).

**TABLE 4:** DIS scores related to quality of life^a^.

	Continued 320 μg/mL glycopyrronium (*n =* 37 enrolled, *n =* 33 completed)	Switched to 320 μg/mL glycopyrronium (*n =* 37 enrolled, *n =* 32 completed)
DIS item 9 score: To what extent did your child's drooling affect his or her life?^a^		
Day 0, median (Q1, Q3)	8.0 (5.0, 9.0)	8.0 (6.0, 10.0)
Day 252, median (Q1, Q3)	1.0 (1.0, 4.0)	2.0 (1.0, 5.0)
Median change on day 252 vs. day 0 (Q1, Q3)	−4.0 (−7.0, −1.0)	−4.0 (−7.0, 0)
*p*	< 0.001	< 0.001
Day 84, median (Q1, Q3)	2.0 (1.0, 5.0)	6.0 (2.0, 9.0)
Day 252, median (Q1, Q3)	1.0 (1.0, 3.0)	2.0 (1.0, 5.0)
Median change on day 252 vs. day 84 (Q1, Q3)	0 (−1.0, 0)	−1.0 (−5.0, 0)
*p*	0.073	< 0.001
DIS item 10 score: To what extent did your child's dribbling affect you and your family's life?^a^		
Day 0, median (Q1, Q3)	9.0 (7.0, 10.0)	9.0 (7.0, 10.0)
Day 252, median (Q1, Q3)	3.0 (1.0, 5.0)	4.0 (1.0, 9.0)
Median change on day 252 vs. day 0 (Q1, Q3)	−5.0 (−7.0, −2.0)	−3.0 (−6.0, 0)
*p*	<0.001	< 0.001
Day 84, median (Q1, Q3)	4.0 (1.0, 7.0)	8.0 (5.0, 10.0)
Day 252, median (Q1, Q3)	3.0 (1.0, 5.0)	4.0 (1.0, 7.0)
Median change on day 252 vs. day 84 (Q1, Q3)	0 (−1.0, 0)	−2.0 (−5.0, 0)
*p*	0.121	< 0.001

Abbreviations: DIS, Drooling Impact Scale; Q, quartile.

^a^ Higher scores indicate greater severity and impact. Children with measures at each time point were analysed.

The number of parents or caregivers fully completing the DISABKIDS‐37 questionnaire on both day 0 and day 252 was low (13 respondents in the continued 320 μg/mL glycopyrronium group and 17 in the switched 320 μg/mL glycopyrronium group). There was no significant difference between the day 0 and day 252 scores in the continued 320 μg/mL glycopyrronium group (0; −6.3, 1.3; *p =* 0.519) and in the switched 320 μg/mL glycopyrronium group (0.6; −4.2, 7.5; *p =* 0.927). The number of children fully completing the DISABKIDS short questionnaire on day 0 and on day 252 was extremely low (three respondents in the continued 320 μg/mL glycopyrronium group and two in the switched 320 μg/mL glycopyrronium group) and precluded statistical analysis of the results.

Between day 0 and day 252, adverse events and treatment‐related adverse events were reported in 86.5% and 51.4% respectively of children who received 320 μg/mL glycopyrronium throughout (Table 5). Between day 84 and day 252, adverse events were reported in 51.4% of children who continued on 320 μg/mL glycopyrronium and 86.5% who switched to 320 μg/mL glycopyrronium. Treatment‐related adverse events between day 84 and day 252 were reported in 13.5% of children in the continued 320 μg/mL glycopyrronium group and 56.8% in the 320 μg/mL switched glycopyrronium group, most commonly constipation (10.8% and 32.4% respectively) and dry mouth (2.7% and 10.8% respectively). These two treatment‐related adverse events were also most common in children who received 320 μg/mL glycopyrronium over the 36‐week period, occurring at a rate of 29.7% for constipation and 10.8% for dry mouth. As observed in the blinded period, there were more adverse events during the initial phase than after long‐term treatment. In total, 15 children (40.9%) who switched to the 320 μg/mL glycopyrronium group experienced at least one treatment‐related adverse event during the 4‐week titration period. After this, 12 patients (32.4%) experienced at least one treatment‐related adverse event during the following 20 weeks. There were no serious treatment‐related adverse events in either group from day 0 to day 252. In the OLSE, non‐serious adverse events led to discontinuation in two children (5.4%) in the continued 320 μg/mL glycopyrronium group (one child had diarrhoea and one had anxiety) and four children (10.8%) in the switched 320 μg/mL glycopyrronium group (one child had seizures; one had aggression, agitation, and faecaloma; one had diarrhoea; and one had rhinitis and impetigo). One additional child, previously in the placebo group, withdrew because of an adverse event (constipation) that occurred before they received 320 μg/mL glycopyrronium and this adverse event was not included in the safety analysis.

**TABLE 5:** Overview of adverse events according to time period.

Children experiencing at least one adverse event, *n* (%)	Day 0–252	Day 84–252	
	Continued 320 μg/mL glycopyrronium (*n =* 37 enrolled, *n =* 33 completed)	Continued 320 μg/mL glycopyrronium (*n =* 37 enrolled, *n =* 33 completed)	Switched to 320 μg/mL glycopyrronium (*n =* 37 enrolled, *n =* 32 completed)
Adverse event	32 (86.5)	19 (51.4)	32 (86.5)
Adverse event leading to treatment discontinuation	2 (5.4)	2 (5.4)	4 (10.8)
Serious adverse event	6 (16.2)	2 (5.4)	3 (8.1)
Treatment‐related adverse event	19 (51.4)	5 (13.5)	21 (56.8)
Most frequent treatment‐related adverse events (occurring in ≥ 2 children [5%] in any treatment arm)			
Constipation	11 (29.7)	4 (10.8)	12 (32.4)
Dry mouth	4 (10.8)	1 (2.7)	4 (10.8)
Urinary retention	1 (2.7)	0 (0)	3 (8.1)
Irritability	2 (5.4)	0 (0)	0 (0)
Psychomotor hyperactivity	2 (5.4)	0 (0)	0 (0)
Treatment‐related adverse event leading to treatment discontinuation	2 (5.4)	2 (5.4)	4 (10.8)
Treatment‐related serious adverse event	0 (0)	0 (0)	0 (0)

## DISCUSSION

Results from the entire 36‐week study period of the SALIVA trial confirm and expand findings from the 3‐month blinded randomized period.^13^ Reductions in total DIS score were sustained over the 36‐week period, such that the significant decrease observed in the first 12 weeks was extended, with a further significant decrease in the subsequent 24 weeks. More than 80% of children experienced a response as defined by a 13.6‐point or greater DIS improvement over the 36‐week period. Indeed, the median 39‐point DIS reduction would be classed as ‘very good to excellent improvement’ according to the carer's global rating of change described by Reid et al.^19^ For children who switched from placebo to 320 μg/mL glycopyrronium on day 84, DIS reductions were observed over the 24‐week OLSE that are consistent with those seen in the active treatment group in the 12‐week randomized blinded phase (median of 66.5 on day 0, reduced to 40 on day 84).^13^ Switching to 320 μg/mL glycopyrronium for 20 weeks enabled an additional 59.5% of children to experience a response.

In this population of children with chronic neurodisabilities and severe sialorrhoea, over one‐third had not received prior pharmacological treatment, which suggests that 320 μg/mL glycopyrronium is effective as a first‐line therapy. In addition, the population included children with drooling that was refractory to other pharmacological therapies, who had been suffering for some years since prior discontinuation, suggesting that 320 μg/mL glycopyrronium treatment is also beneficial in previously treated patients. Baseline data also highlight that there is no clear treatment strategy for severe drooling in children with chronic neurodisabilities in France given the range of previous treatments reported, which are often used off‐label. Guidelines concerning the treatment of drooling in children with chronic neurodisabilities are only available in some countries but would help to guide management decisions now that there are licensed agents—glycopyrronium and botulinum neurotoxin A—and trial evidence available.

The impact of drooling on the child's and family's lives was reduced with 320 μg/mL glycopyrronium over the 36‐week period. In the population who had already received 320 μg/mL glycopyrronium, numerical reductions continued to be observed on the impact of drooling on the child's and family's lives from day 84 to day 252, albeit not statistically significant, having already been reduced in the blinded phase of the study. As expected, the impact of drooling on the lives of children and their families was significantly reduced when children were switched from placebo to 320 μg/mL glycopyrronium. Although these findings are based on isolated items from the DIS questionnaire, they were analysed as prespecified endpoints and provide important information on the treatment's effect on the impact of drooling on QoL, an area that has been understudied to date. Parents readily answered the drooling‐specific QoL questions in the DIS‐F questionnaire but were much more reticent to fully complete the DISABKIDS questionnaire, which explored whether an improvement in drooling could be measured in terms of overall QoL. Full completion rates of the DISABKIDS questionnaires, designed for children with chronic diseases in general, were even lower than observed in the randomized period. The number of questions may have been burdensome, the questions themselves may not have been thought appropriate, or they may have been difficult to answer with regard to children with neurodisabilities. Many questions were left without a response, particularly in areas related to ‘mental emotion (inner strength)’, ‘social inclusion’, and ‘physical treatment’ in both groups and at each assessment. Further tools to assess QoL in children with neurodisabilities, across the age range from 3 to 18 years, are warranted, although this may be complicated by different presentations and degrees of severity.

A key aim of the OLSE was to assess the long‐term safety profile of 320 μg/mL glycopyrronium, particularly as the longest trial conducted with glycopyrronium (a 160 μg/mL formulation) lasted only 24 weeks.^16^ Fewer children experienced treatment‐related adverse events (13.5%) and discontinued because of treatment‐related adverse events (5.4%) with 36 weeks of continued 320 μg/mL glycopyrronium than after switching to 320 μg/mL glycopyrronium for 24 weeks (56.8% and 10.8%), which probably reflects a reduction in adverse events after the first few weeks of titration. Twenty‐four‐week results in children who switched to 320 μg/mL glycopyrronium are consistent with 12‐week findings from the blinded period.^13^

In common with other anticholinergics and studies with glycopyrronium,^7^, ^15^, ^21^, ^22^ constipation and dry mouth were the most frequent adverse events, but frequency was lower with long‐term use (36 weeks: 10.8% and 2.7% respectively) than after switched treatment in the OLSE (24 weeks: 32.4% and 10.8% respectively) and in the blinded phase (12 weeks: 20.5% and 6.8% respectively). Other treatment‐related adverse events identified as common (> 10%) in the formulation's Summary of Product Characteristics, such as irritability, flushing, vomiting, and urinary retention^10^ occurred less frequently over the 36‐week period. This may be because of slow dose titration over a period of up to 4 weeks, allowing an effective and well‐tolerated dose to be reached, which may not be routine with anticholinergics in clinical practice. Additional factors may include the ability to administer the dose accurately with the oral formulation, limited ability to titrate the dose in previous studies, or the nature of adverse event collection in other trials.

Limited trial data are available comparing different anticholinergics; however, glycopyrronium was associated with better tolerability than other agents in the 12‐week open‐label Drooling Reduction Intervention trial assessing 160 μg/mL glycopyrronium (1 mg/5 mL glycopyrronium bromide) and scopolamine patches^15^ and in a real‐world study of glycopyrronium (unknown off‐label formulation), benzhexol (trihexyphenidyl), and scopolamine of up to 52 weeks' duration.^21^ In the Drooling Reduction Intervention trial (*n* = 90), 36% of parents reported a predictable side effect that led to cessation of scopolamine (11 with skin reactions, one with dry mouth, one with pupil dilation; four children repeatedly pulled off their patches) compared with 16% with glycopyrronium (two with dry mouth, two with constipation, and two with skin dryness or rash).^15^

The 320 μg/mL glycopyrronium dose used remained relatively stable with long‐term use, was in the range between dose levels 3 and 4, and was consistent with the dose per weight used in other trials.^14^, ^15^ Some volume increases and decreases were observed, as expected with anticholinergics, where the dose can be affected by several factors, including weight, age, the weather, and physical activity. It is recommended that after the initial dose titration period of treatment, the child's sialorrhoea is monitored in conjunction with the parent or carer at no longer than 3‐month intervals, to assess changes in efficacy or tolerability over time, and the dose adjusted accordingly.

Regarding limitations, the trial excluded children who had not received at least 3 months of non‐pharmacological oral rehabilitation. Despite being the standard of care based on French guidelines,^17^ most children seen in clinical practice in France do not receive oral rehabilitation. The modified Teacher's Drooling Scale was used to assess eligibility because it includes a definition for ‘severe’ and 320 μg/mL glycopyrronium is indicated for severe sialorrhoea; however, a French version of the DIS, which was approved by the Mapi Institute and a DIS author, was chosen as the most appropriate scale to measure the effect of drooling over time. Both DIS and the DIS‐F versions have been used in other studies on drooling^15^, ^19^ and are the only evaluative tools with responsiveness data that are useful for detecting clinically important changes over time.^23^ Furthermore, an evaluation of the DIS, modified Teacher's Drooling Scale, and the Drooling Severity and Frequency Scale found that while all were effective in the diagnosis of drooling, both for rating its severity and frequency, DIS was the only tool that considered the physical complications of drooling and their impact on QoL.^24^

## CONCLUSIONS

In children with neurodisabilities and severe sialorrhoea, long‐term treatment with a paediatric‐specific formulation of 320 μg/mL glycopyrronium resulted in sustained improvements in drooling over 36 weeks and reductions in the impact of drooling on QoL, with fewer adverse events after initial titration and overall good tolerability.

## FUNDING INFORMATION

Proveca Ltd. funded the trial. In the drafting of the manuscript, editorial assistance was provided by Emma Marshman and funded by Proveca Ltd.

## CONFLICT OF INTEREST STATEMENT

PF receives fees from Merz Pharma for providing training sessions. HS and FV are employees of Proveca Ltd. GL has given lectures for Nutricia Clinical Nutrition and Nestlé Health Science. SA has served as consultant or given lectures for Angelini Pharma, Biocodex, Eisai, Encoded Therapeutics, Grintherapeutics, Jazz Pharmaceuticals, Neuraxpharm, Orion, Nutricia, Proveca, UCB Pharma, Vitaflo, Xenon, and Zogenix. SA has been an investigator on clinical trials for Eisai, Jazz Pharmaceuticals, Marinus Pharmaceuticals, Proveca, Takeda Pharmaceuticals, and UCB Pharma. DS is an employee of the contract research organization, Kappa Santé. MD and VG report no competing interests.

## Supporting information


**Figure S1:** Trial design overview.

**Figure S2:** Consolidated Standards of Reporting Trial diagram.

**Table S1:** Dosing table used in the SALIVA trial (all participants had normal renal function)

## Data Availability

The dataset analyzed during the current study are not publicly available due to health data protection but are available from the corresponding author on reasonable request.
