# Supplementary material for: Glycopyrronium 320 μg/mL in children and adolescents with severe sialorrhoea and neurodisabilities: An open‐label study extension of the SALIVA trial
Source: Dev Med Child Neurol. 2025 Jan 31;67(8):1085–94. doi: 10.1111/dmcn.16251 (PMC12237225; doi:10.1111/dmcn.16251)
Supplement: Supplementary file 1 — Figure S1: Trial design overview. [file DMCN-67-1085-s003.docx]

**FIGURE S1 Trial design overview.**

**
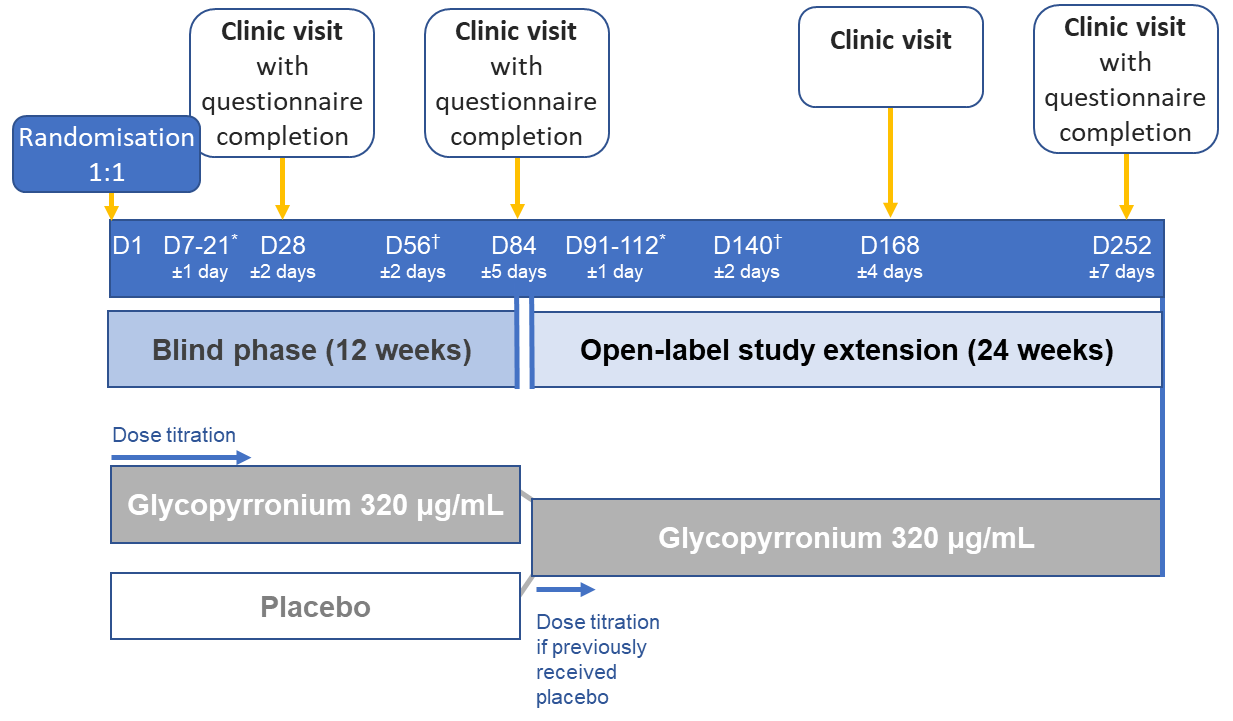
**

^*^Telephone interviews once a week
^†^Telephone interview

**Dose titration**

The dose of study drug was titrated over a period of up to 4 weeks consistent with the Summary of Product Characteristics of the licensed drug for children and adolescents with normal renal function (Table S1).^1^ Participants received their first dose according to their weight three times per day and increased the dose every 7 days using specific dose levels. Dose titration was continued until efficacy was balanced with any undesirable effects and the dose was amended up or down as appropriate to a maximum individual dose.
