# Supplementary material for: Glycopyrronium 320 μg/mL in children and adolescents with severe sialorrhoea and neurodisabilities: An open‐label study extension of the SALIVA trial
Source: Dev Med Child Neurol. 2025 Jan 31;67(8):1085–94. doi: 10.1111/dmcn.16251 (PMC12237225; doi:10.1111/dmcn.16251)
Supplement: Supplementary file 2 — Figure S2: Consolidated Standards of Reporting Trial diagram. [file DMCN-67-1085-s002.docx]

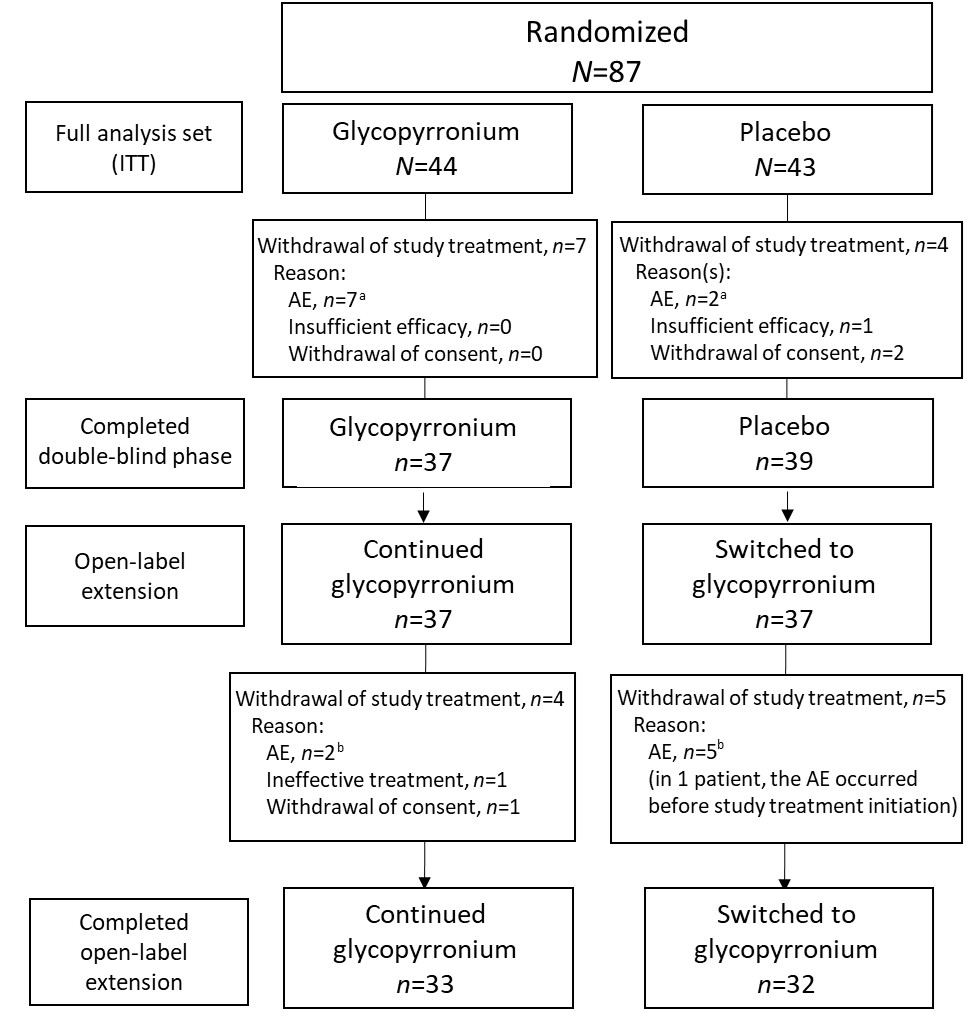


**Figure S2:** Consolidated Standards of Reporting Trial diagram. ^a^Treatment-related adverse events (AEs) led to study treatment discontinuation in 7 participants in the 320μg/ml glycopyrronium group: abdominal pain and constipation (1 patient, considered a serious AE); vomiting and drug intolerance (1 patient); flushing and nervousness (1 patient); seizure, vomiting, visual impairment, and decreased appetite (1 patient each). Two AEs led to treatment discontinuation in two participants in the placebo group (fatigue and salivary hypersecretion). ^b^Treatment-related AEs led to study treatment discontinuation in 2 participants in the continued 320μg/ml glycopyrronium group: 1 participant had diarrhoea and 1 had anxiety. Treatment-related AEs led to treatment discontinuation in 4 participants in the switched 320μg/ml glycopyrronium group: 1 had seizures; 1 had aggression, agitation, and faecaloma; 1 had diarrhoea; and 1 had rhinitis and impetigo. One participant in the switched group (previously on placebo) had an AE before the initiation of study treatment in the open-label study extension (constipation).
