# Supplementary material for: Glycopyrronium 320 μg/mL in children and adolescents with severe sialorrhoea and neurodisabilities: An open‐label study extension of the SALIVA trial
Source: Dev Med Child Neurol. 2025 Jan 31;67(8):1085–94. doi: 10.1111/dmcn.16251 (PMC12237225; doi:10.1111/dmcn.16251)
Supplement: Supplementary file 3 — Table S1: Dosing table used in the SALIVA trial (all participants had normal renal function) [file DMCN-67-1085-s001.docx]

**Table S1.** **Dosing table used in the SALIVA trial (all participants had normal renal function)**^†^

| Weight | Dose level 1 | Dose level 2 | Dose level 3 | Dose level 4 | Dose level 5 |
| --- | --- | --- | --- | --- | --- |
|  | **~12.8 μg/kg**^‡^ | **~25.6 μg/kg**^‡^ | **~38.4 μg/kg**^‡^ | **~51.2 μg/kg**^‡^ | **~64.0 μg/kg**^‡^ |
| kg | **mL** | **mL** | **mL** | **mL** | **mL** |
| 13–17 | 0.6 | 1.2 | 1.8 | 2.4 | 3.0* |
| 18–22 | 0.8 | 1.6 | 2.4 | 3.2 | 4.0* |
| 23–27 | 1.0 | 2.0 | 3.0 | 4.0 | 5.0* |
| 28–32 | 1.2 | 2.4 | 3.6 | 4.8 | 6.0* |
| 33–37 | 1.4 | 2.8 | 4.2 | 5.6 | 6.0* |
| 38–42 | 1.6 | 3.2 | 4.8 | 6.0* | 6.0 |
| 43–47 | 1.8 | 3.6 | 5.4 | 6.0* | 6.0 |
| ≥48 | 2.0 | 4.0 | 6.0* | 6.0 | 6.0 |

^†^An additional dosing table is available in the Summary of Product Characteristics^1^ for children and adolescents with mild to moderate renal impairment (all participants had normal renal function in the SALIVA trial)
^‡^Refers to μg/kg glycopyrronium
***Maximum individual dose in this weight range

**Reference**

1. European Medicines Agency. Sialanar. Available at: www.ema.europa.eu/en/medicines/human/EPAR/sialanar (accessed 18 March 2024).
